# Evaluation of Vegetation Index-Based Curve Fitting Models for Accurate Classification of Salt Marsh Vegetation Using Sentinel-2 Time-Series

**DOI:** 10.3390/s20195551

**Published:** 2020-09-28

**Authors:** Chao Sun, Jialin Li, Luodan Cao, Yongchao Liu, Song Jin, Bingxue Zhao

**Affiliations:** 1 Department of Geography & Spatial Information Techniques, Ningbo University, Ningbo 315211, China; lijialin@nbu.edu.cn (J.L.); caoluodan@nbu.edu.cn (L.C.); 2 Key Laboratory of Coastal Zone Exploitation and Protection, Ministry of Natural Resources, Nanjing 210023, China; yongchaoliu@smail.nju.edu.cn (Y.L.); jinsong@smail.nju.edu.cn (S.J.); bingxue@smail.nju.edu.cn (B.Z.); 3 Institute of East China Sea, Ningbo University, Ningbo 315211, China; 4 School of Geography and Ocean Science, Nanjing University, Nanjing 210023, China; 5 School of Geography and Planning, Chizhou University, Chizhou 247000, China

**Keywords:** Sentinel-2 imagery, time series, salt marsh, classification mapping, vegetation index, curve fitting method

## Abstract

The successful launch of the Sentinel-2 constellation satellite, along with advanced cloud detection algorithms, has enabled the generation of continuous time series at high spatial and temporal resolutions, which is in turn expected to enable the classification of salt marsh vegetation over larger spatiotemporal scales. This study presents a critical comparison of vegetation index (VI) and curve fitting methods—two key factors for time series construction that potentially influence vegetation classification performance. To accomplish this objective, the stability of five different VI time series, namely Normalized Difference Vegetation Index (NDVI), Soil-Adjusted Vegetation Index (SAVI), Enhanced Vegetation Index (EVI), Green Normalized Difference Vegetation Index (GNDVI), and Water-Adjusted Vegetation Index (WAVI), was compared empirically; the suitability between three curve fitting methods, namely Asymmetric Gaussian (AG), Double Logistic (DL), and Two-term Fourier (TF), and VI time series was measured using the coefficient of determination, and the salt marsh vegetation separability among different combinations of VI time series and curve fitting methods (i.e., VI time series-based curve fitting model) was quantified using overall the Jeffries–Matusita distance. Six common types of salt marsh vegetation from three typical coastal sites in China were used to validate these findings, which demonstrate: (1) the SAVI performed best in terms of time series stability, while the EVI exhibited relatively poor time series stability with conspicuous outliers induced by the sensitivity to omitted clouds and shadows; (2) the DL method commonly resulted in the most accurate classification of different salt marsh vegetation types, especially when combined with the EVI time series, followed by the TF method; and (3) the SAVI/NDVI-based DL/TF model demonstrated comparable efficiency for classifying salt marsh vegetation. Notably, the SAVI/NDVI-based DL model performed most strongly for high latitude regions with a continental climate, whilst the SAVI/NDVI-based TF model appears to be better suited to mid- to low latitude regions dominated by a monsoonal climate.

## 1. Introduction

Salt marshes are flat, muddy coastal wetlands which are periodically flooded by tides and are widely accepted as one of the most productive and valuable ecosystems in the world [1]. As the primary producer of these ecosystems, salt marsh vegetation provides several important ecological services such as sediment trapping, water filtration, wildlife shelter, nutrient cycling, and carbon sequestration [2]. It also serves as a vital indicator of salt marsh stability, as net erosion or deposition changes the local topography and tidal regime, which in turn affects the edaphic conditions and vegetation composition [3,4]. Currently, the acquisition of accurate information on salt marsh vegetation species composition and distribution is urgently needed given that a significant number of salt marshes worldwide are being destroyed by climate change and anthropogenic disturbances [5,6].

Remote sensing has several major advantages over labor-intensive field-based surveys, namely, its synoptic coverage and repeatability. For classification mapping of salt marsh vegetation from an individual image, previous studies have tended to deploy high-spatial or hyper-spectral resolution data given the high similarity in spectral signatures among different salt marsh vegetation types [7,8,9,10,11,12]. However, the ability of these data to effectively differentiate salt marsh vegetation is offset by their paucity, relatively high cost, and that they are restricted to small areas and brief time intervals. To mitigate these limitations, the possibility of discriminating between different types of salt marsh vegetation using a remotely sensed time series approach has been confirmed by multi-temporal field spectra collections [13,14]. However, determination of the suitable balance between temporal and spatial resolution for time series construction remains challenging. The traditional vegetation index (VI) composites (e.g., MODIS NDVI/EVI, AVHRR GVI) possess a coarse spatial resolution (>250 m), rendering them unsuitable for salt marsh regions characterized by a narrow distribution and high degree of heterogeneity [15,16,17]. Alternatively, the time interval of cloud-free moderate spatial resolution imagery (e.g., Landsat TM/ETM + /OLI, HJ CCD) usually exceeds one month, which is unlikely to adequately characterize the variability of salt marsh vegetation over time [18,19,20].

Recently, with the successful launch of the Sentinel-2 A/B constellation satellite in July 2017, optical images with a temporal resolution of five days have been made available [21,22,23]. On the other hand, the availability of increasingly accurate cloud detection algorithms has provided opportunities to make full use of all of optical imagery instead of only cloud-free images [24]. These developments have facilitated the construction of time series with a temporal resolution comparable to traditional MODIS NDVI/EVI composites, but with enhanced spatial resolution (e.g., up to 10 m). Such time series methods have been increasingly applied to agricultural, forested, and wetland ecosystems with high success rates [4,16,17,21,25]. Notably, the irregular appearance of clouds and their shadows from different acquisition times determines the different distributions of available observations for each pixel in the time series (hereafter referred to as “pixel-differential time series”). For time series based solely on cloud-free images, the VI values from each image are used as classification features because of the uniform distribution of the available observations. However, in terms of pixel-differential time series, the classification features of each pixel principally derive from several phenological parameters in a fitting curve [26,27], which is generated according to the observed distribution of specific VI values.

Selections of VI and fitting curves are two key determinants governing the final performance of salt marsh vegetation classification within complex coastal environments. Firstly, periodic tidal flooding hinders remote sensing of salt marsh vegetation by obscuring spectral reflectance and introducing noise. Such noise caused by water and moist soil has been reported to differentially attenuate the intensity of the visible and near-infrared spectrum [28]. In addition, variations in the tidal regime introduce fluctuations in time series data as images capture different degrees of tidal flooding. Hence, selecting the VI which is least sensitive to tidal flooding and which preserves the general trend of pixel-differential time series is essential. Secondly, cloud cover also prohibits remote sensing of salt marsh vegetation. Although this effect can be mitigated to a large degree by cloud detection algorithms, there is still a loss of observational data [29,30]. More importantly, unlike traditional MODIS NDVI/EVI composites, where the observation in the time series is distributed at equal intervals, the distribution of the pixel-differential time series is sparse, uneven, and may fluctuate in response to local climate factors. As such, it is important to select suitable fitting curves to adequately match the distribution of the pixel-level differential time series.

In summary, although the application of pixel-differential time series methods to the analysis of salt marsh vegetation classification shows much promise, comparisons and evaluations of the various VIs, curve fitting methods, and their combinations (i.e., VI time series-based curve fitting models) are still needed. Compared to previous studies [31,32,33,34], the potential value of our study is threefold: (1) most of the data previously used for time series construction were MODIS NDVI/EVI products, where the distribution of available observations for each pixel was even (e.g., 16 days, one month); notably in the present study, the distribution of observations from the time series was derived from Sentinel-2 imagery; (2) previous studies primarily used inland forest ecosystems owing to relatively favorable imaging conditions; in contrast, we use salt marsh ecosystems located in coastal areas, in order to devise a new approach to overcome the limitations imposed by frequent cloudy weather and periodic tidal fluctuations on the acquisition and quality of satellite imagery; and (3) most previous studies discussed the accuracy from the different curve fitting methods without considering the suitability of the VI time series, and whether high accuracy can guarantee the success for subsequent application of time series analyses (e.g., classification mapping) was also mentioned. Therefore, the specific aims of this paper are to: (1) compare the stability among different VI time series, (2) measure the suitability between different curve fitting methods and VI time series; and (3) determine which of the VI time series-based curve fitting models perform best for discriminating salt marsh vegetation types. To address these aims, six common salt marsh vegetation types from three typical coastal sites in China were analyzed. Five VIs and three curve fitting methods were selected for comparison as the most common approaches currently available.

## 2. Materials

### 2.1. Study Sites

**Figure 1:**
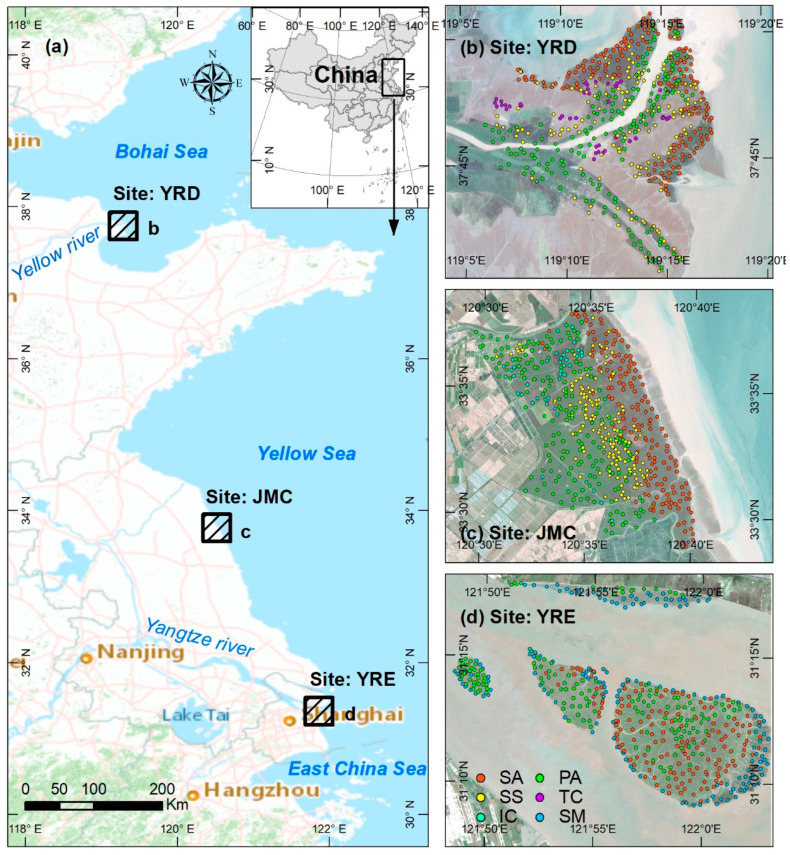
(**a**) Location map showing the three study sites, the Yellow River Delta (YRD), Jiangsu Middle Coast (JMC), and Yangtze River Estuary (YRE); (**b**–**d**) enlarged Sentinel-2 images of each study area highlighting the distribution of six dominant salt marsh vegetation samples in the YRD, JMC, and YRE. Note: SA = *S. alterniflora*, PA = *P. australis*, SS = *S. salsa*, TC = *T. chinensis*, IC = *I. cylindrica*, and SM = *S. mariqueter*.

Three typical coastal sites in China were studied, each of which covered a 350 km^2^ area (Figure 1a), summarized as follows: (1) Yellow River Delta (YRD), which lies in the estuary of the Yellow River, which exhibits some of the highest global sediment yields; (2) Jiangsu Middle Coast (JMC), where long-shore drift over the past several centuries has reworked ancient sediments from the ancient Yellow River and Yangtze River; (3) Yangtze River Estuary (YRE), which consists of several offshore sandbars formed by fluvial material from the Yangtze River.

Each of the three study sites are characterized by different hydroclimatic conditions and tidal regimes. The YRD is located within a semi-arid area, subjected to a temperate continental monsoon climate with an average temperature of 11.9 °C and annual precipitation of ~580 mm. Here, there is a mild tidal effect, with an average tidal range of 0.5 m and a maximum range of 1.7 m. The JMC is located in the warm-temperate zone and is subjected to a marine monsoon climate with an average temperature of 14.5 °C and annual precipitation of ~970 mm. There are greater tidal fluctuations in the JMC, with an average tidal range of 1.9 m and a maximum tidal range of 3.3 m. The YRE has a subtropical monsoon climate with an average temperate of 16 °C. The annual precipitation is 1150 mm, with 60% of rainfall occurring from May to September. The YRE is profoundly influenced by tidal currents, with an average tidal range of 2.7 m and a maximum 4.7 m.

The broad tidal flats of the three study sites are occupied by several dominant native salt marsh vegetation types—*Phragmites australis* (*P. australis*), *Tamarix Chinensis* (*T. chinensis*), and *Suaeda salsa* (*S. salsa*) in the YRD (Figure 1b); *Imperata cylindrica* (*I. cylindrica*), *P. australis*, and *S. salsa* in the JMC (Figure 1c); *P. australis* and *Scirpus mariqueter* (*S. mariqueter*) in the YRE (Figure 1d). The invasive species *Spartina alterniflora* (*S. alterniflora*), has rapidly spread along Chinese coasts after its introduction in 1978 and has become the dominant salt marsh vegetation type in the three study areas due to its strong adaptability and productivity [35]. Currently, *S. alterniflora* has widely spread in each study site and has become another dominant salt marsh vegetation type.

### 2.2. Sentinel-2 MSI Images

**Figure 2:**
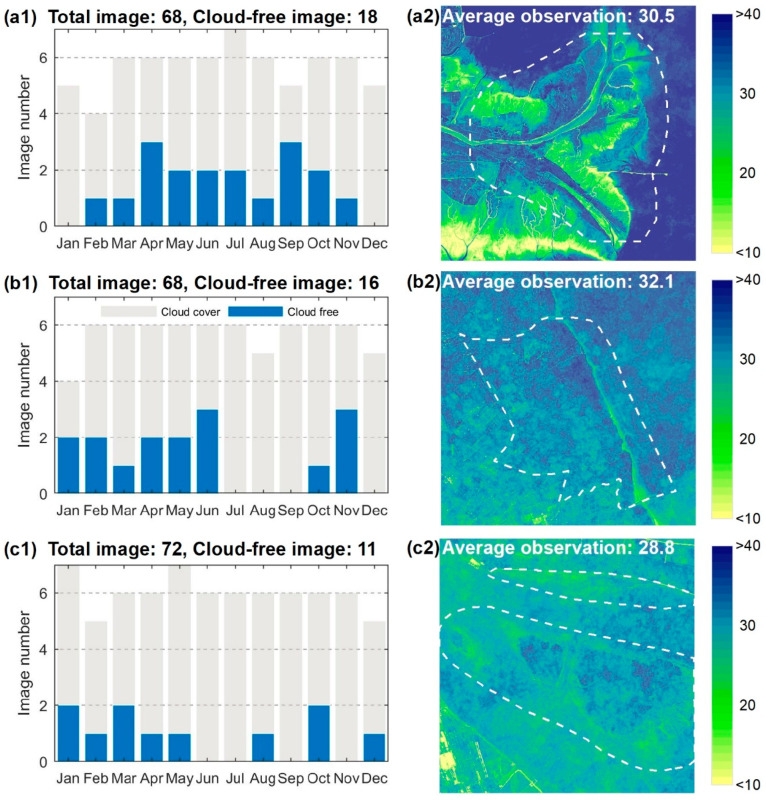
Improvements in the number of available observations from the pixel-differential time series. (**a1**–**c1**) Temporal distribution patterns of available observations from cloud-free images for the YRD, JMC, and YRE, respectively; (**a2**–**c2**) spatial distribution pattern of available observations from the pixel-differential time series for the YRD, JMC, and YRE, respectively.

The Sentinel-2 A/B constellation includes two identical satellites operating in a sun-synchronous orbit, enabling an approximate 5-day revisit time [21]. The Sentinel-2 A/B MSI records the Earth reflected radiance in 13 spectral bands covering the Visible and Near-Infrared (VNIR) to Short-Wave Infrared (SWIR) spectrum with different spatial resolutions (10, 20, and 60 m, respectively). We downloaded Sentinel-2 MSI Level-1C (L1C) images from the European Space Agency (ESA) Sentinel Scientific Data Hub. These images provide Top-Of-Atmosphere (TOA) reflectance and are assigned as 100 km tiles by preliminary radiometric and geometric corrections. All the Sentinel-2 MSI L1-C images covering the three study sites during 2018 were collected without considering cloud cover. These included 68 tiles for the YRD (Figure 2(a1)), 68 tiles for the JMC (Figure 2(b1)), and 72 tiles for the YRE (Figure 2(c1)). These images were used to construct pixel-differential time series in order to compare the performance of the different VIs and fitting curves.

### 2.3. Saltmarsh Vegetation Samples

**Figure 3:**
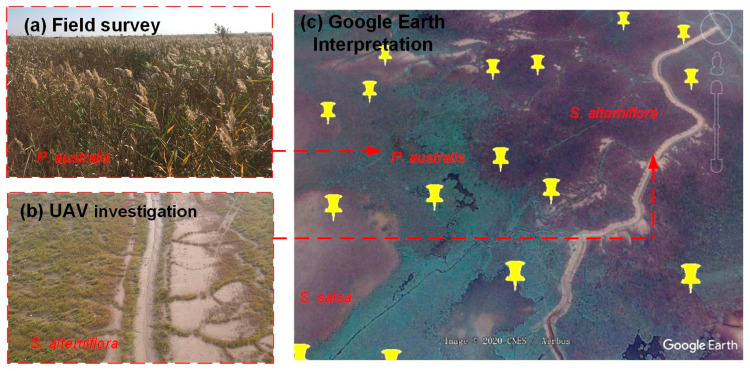
Sample collection methods employed in salt marsh ecosystems, including (**a**) field survey, (**b**) UAV investigation, and (**c**) Google Earth imagery. Note: red arrows point to photograph locations obtained using both a handheld camera and UAV survey, and the yellow pins represent samples randomly generated in ArcGIS.

A total of 1502 samples (Figure 1b–d) were collected using three methods in order to measure the separability among different salt marsh vegetation types, summarized in Table 1 and Figure 3. (1) Field surveys were conducted from April 6 to November 29, 2018, at each study site, and the six dominant salt marsh vegetation types were sampled. A total of 186 samples were surveyed, most of which were located nearby roads and dams; (2) An Unmanned Aerial Vehicle (UAV) investigation was carried out together with the field surveys, in order to generate synoptic information pertaining to salt marsh vegetation in inaccessible locations, from which 406 samples were collected in close proximity (~3 km) to the samples obtained during the field survey; (3) Google Earth imagery was used to provide an additional 910 samples and ensured complete representation of the distribution of salt marsh vegetation across each of the three study areas. These samples were randomly generated using ArcGIS software (version: 10.3) and labeled with specific salt marsh vegetation information by interpreting high-resolution snapshots from Google Earth.

## 3. Methods

### 3.1. Pixel-Differential Time-Series Construction

**Figure 4:**
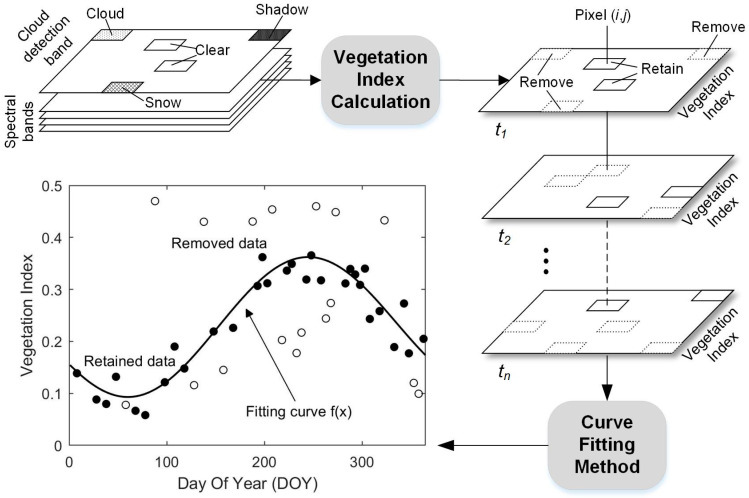
Summary of pre-processing methods used in the construction and application of pixel-differential time series analyses.

Atmospheric corrections were implemented across all Sentinel-2 MSI L1C images to convert TOA to Bottom-Of-Atmosphere (BOA) reflectance using the Sen2Cor processor (Version 2.8.0). Four bands (2, 3, 4, and 8) at a spatial resolution of 10 m were introduced in this process for the consideration of the high spatial heterogeneity of the salt marsh vegetation. Secondly, to minimize atmospheric effects, clouds, cloud shadows, and snow pixels for each image were identified and removed using the Scene Classification (SC) map (Figure 4). Specifically, the pixels of the SC map which exhibited values of 3 (cloud shadow), 8–10 (cloud/cirrus with different confidence), and 11 (snow) were removed. Finally, the remote-sensing-based Vegetation Index (VI) of each image was calculated and sorted into chronological order to construct a pixel-differential time series. Compared with the time series based on cloud-free images, the available observations using the pixel-level time series were significantly improved (Figure 2(a2–c2)).

### 3.2. Vegetation Index Selection

The utility of the VI is well documented in relation to time series construction because the spectral band combinations are more sensitive to different vegetation characteristics (e.g., structure, pigments, and water content) than individual bands. The Normalized Difference Vegetation Index (NDVI) is easily calculated using red and near-infrared bands available with most optical sensors and has been widely used to describe vegetation phenology. Several modified VIs have been subsequently designed to factor in the influences from different backgrounds by introducing additional non-vegetation-related bands and adjustment factors into NDVI. For example, the Enhanced Vegetation Index (EVI) was created to enhance the vegetation signal in high biomass regions through a de-coupling of the canopy background signal and a reduction in atmosphere influences. The Green Normalized Difference Vegetation Index (GNDVI) is related to the proportion of photosynthetically absorbed radiation and exhibits a linear correlation with biomass, rendering it more sensitive to chlorophyll concentration than NDVI. The Soil-Adjusted Vegetation Index (SAVI) minimizes the influence of soil brightness by employing a canopy background adjustment factor (*L*), which is suitable for areas where vegetation distribution is scattered and the soil surface is exposed. The Water-Adjusted Vegetation Index (WAVI) is designed for capturing aquatic vegetation characteristics and distinguishing aquatic from terrestrial vegetation via the integration of the spectral response in the shorter visible wavelength range.

The above five VIs were chosen based on full consideration of the characteristics of salt marsh vegetation. Substantial variations in plant density among different salt marsh vegetation species were observed during our field surveys, ranging from >100 plants/m^2^ (e.g., *S. alterniflora*, *P. australis*) to <20 plants/m^2^ (e.g., *T. chinensis*, *S. salsa*), thereby necessitating that the impacts of vegetation saturation and soil background be considered [36]. Besides, given that salt marsh vegetation is frequently submerged by tides and that pioneer vegetation has a low plant height and density (e.g., *S. mariqueter* in particular), the water-related VI was employed. Another reason for selecting these VIs is because their calculation does not involve Sentinel-2 MSI bands with coarse spatial resolution >10 m. These five VIs were calculated using the formulas in Table 2.

### 3.3. Curve Fitting Method and Comparison

Smoothing methods are typically applied to VI time series data to minimize noise. In particular, curve fitting applies mathematical functions to fit the VI time series to a specified function. This effectively suppresses noise and can be easily adapted to a range of datasets without the need for pre-defined thresholds or empirical constraints [34]. In our study, three curve fitting methods were selected for comparison.

(1) Asymmetric Gaussian (AG)

AG is formed by a piecewise function. Each piece is fitted with a Gaussian function allowing different parameter values [42]. The AG was calculated using Equation (1).

(1) $$f(t)=c_{1}+c_{2}\cdot \{\begin{array}{c} \exp\left[-{\left(\frac{a_{1}-t}{a_{2}}\right)}^{a_{4}}\right],\;t\leq a_{1}\\ \exp\left[-{\left(\frac{t-a_{1}}{a_{3}}\right)}^{a_{4}}\right],\;t>a_{1} \end{array}$$

where *t* is the day of the year, coefficients *c_1_* and *c_2_* represents the base level and amplitude of the function, respectively; the coefficient *a_1_* approximates the position (in time) of the maximum value, coefficients *a_2_* and *a_3_* are related to the width of the left and right half function, and the coefficient *a_4_* is related to the flatness of the function.

(2) Double Logistic (DL)

Similar to the AG, DL determines the final curve function using piecewise local fitting [43], which is calculated using Equation (2).

(2) $$f(t)=c_{1}+c_{2}\cdot \left[\frac{1}{1+\exp\left(\frac{a_{1}-t}{a_{2}}\right)}-\frac{1}{1+\exp\left(\frac{a_{3}-t}{a_{4}}\right)}\right]$$

where *t* is the day of the year, coefficients *c_1_* and *c_2_* represent the base level and amplitude of the function, respectively; coefficients *a_1_* and *a_3_* determine the position of the left and right inflection points of the curve, respectively; and coefficients *a_2_* and *a_4_* determine the rate of change of the left and right inflection points, respectively.

(3) Two-term Fourier (TF)

The TF is comprised of two pairs of sine and cosine functions, which effectively represent inter-annual vegetation changes [44]. The formula of the TF is given in Equation (3).

(3) $$\begin{array}{ll} f(t)=c & +a_{1}\cos(wt)+b_{1}\sin(wt)\\ & +a_{2}\cos(2wt)+b_{2}\sin(2wt) \end{array}$$

where *t* is the day of the year, the coefficient *c* represents the base level of the function, the coefficient of *w* denotes the frequency and approximates 0.017 (2π/365), coefficients *a_1_* and *b_1_* represent general changes in vegetation phenology; and coefficients *a_2_* and *b_2_* allow us to describe local phenological variation of different vegetation types.

The ability to describe VI time series is highly related to the number of coefficients present. Generally, more coefficients enable the fitting curve to better capture subtle variations, as they can describe more detailed changes within the VI time series. Therefore, the format of the above three curve fitting methods we introduced was determined after careful consideration. Specifically, the formats of the AG and DL functions refer to Jonsson and Eklundh [42] and have been widely used in time series analyses in various vegetated ecosystems (e.g., forest, farmland, grassland) for more than one decade. The format of the TF was proposed by Zhu, Woodcock, and Olofsson [44], the function of which has been increasingly used as a benchmark for monitoring real-time changes using Landsat time series in recent years. Besides, six coefficients were used for each of the three curve fitting methods in order to facilitate suitable comparisons between the different fitting results from each of the methods employed. Note that the DL function in another form that employed four coefficients was abandoned in spite of recent wide use [45,46] because it omits the vegetation senescence phase (September to December), which is crucial for identifying salt marsh vegetation, especially *S. alterniflora*.

As a direct index of suitability, the fitting accuracy represented by the coefficient of determination (R^2^) among the different VI time series and curve fitting methods was calculated on the basis of the salt marsh vegetation samples. One-way ANOVA (ANalysis Of VAriance) was performed on the R^2^ among different VI time series and curve fitting methods to determine whether there were any statistically significant differences among the means of classes [47]. Note that one-way ANOVA does not specify which classes are different. Therefore, the Tukey HSD (Honestly Significant Difference) was also introduced in conjunction with the ANOVA to establish which pairs of classes exhibited a significantly different mean value [48].

### 3.4. Phenological Metric Extraction

Based on each fitting curve, six commonly used metrics to describe vegetation phenology were then extracted using the threshold approach which included two value-related metrics (BV and MV), two time-related metrics (SOS and EOS), and two rate-related metrics (ROI and ROD). The calculations for these phenological metrics are listed in Table 3. The threshold values of SOS and EOS were determined by testing continuous discrete values from 10% to 90% at increments of 10%; 50% was finally selected because the time determined by this value exhibited the strongest agreement with the actual status of salt marsh vegetation as observed during the field survey. This value is also encouragingly consistent with the threshold suggested by Eklundh and Jönsson [49]. As such, the two other suggested values (20% and 80%) were accepted to determine the ROI and ROD, which are the respective ratios of the difference in values during either the green-up or senescence season, and the corresponding time difference. We point out that evaluation of the best phenological metric or metric combination for the classification of salt marsh vegetation was not conducted, since extraction of as many phenological metrics as possible rather than a subset is best in order to maximize classification accuracy. Other phenological metrics (e.g., season length, seasonal amplitude, and season mid-point) were not considered as their inclusion resulted in no measurable improvements in separability among different types of salt marsh vegetation owing to their high correlation with the six coefficients outlined earlier.

Owing to the high temporal resolution of the Sentienel-2 images, the majority of pixels (>99.9%) in the study areas had >6 observations after cloud masking (Figure 2(a2–c2)). With respect to the vegetation samples, the number of available observations was never less than 20, which enabled the successful implementation of the curve fitting and subsequent analyses. Note that the very few pixels with less than six available observations mainly originated from tidal flats near sea. In these cases, the curve fitting was abandoned and [0, 0, 0, 0, 0, 0] were directly assigned as the phenological parameters during the process of classification mapping.

### 3.5. Separability Analysis of Salt Marsh Vegetation

The Jeffries–Matusita distance (JMD) was used to quantify the separability among different salt marsh vegetation types. The JMD takes into account the distance between class means and the distribution of values from the means, which is achieved by incorporating the covariance matrices of the classes in the separability measurements [50]. The JMD can be used to measure the pairwise separability between two classes, thereby allowing an assessment of the differences between the selected class samples in the feature space. The JMD between two classes *i* and *j* was calculated using Equation (4).

(4) $$JMD_{ij}=2(1-\exp(-B_{ij}))$$

where *B_ij_* is the Bhattacharyya distance between the two classes *i* and *j* and is defined as Equation (5).

(5) $$B_{ij}=\frac{1}{8}{\left(m_{i}-m_{j}\right)}^{T}{\left[\frac{\sum_{i}+\sum_{j}}{2}\right]}^{-1}(m_{i}-m_{j})+\frac{1}{2}\ln\frac{\left|(\sum_{i}+\sum_{j})/2\right|}{\sqrt{\left|\sum_{i}\right|\left|\sum_{j}\right|}}$$

where *m_i_* and *m_j_* denote the mean values, and *∑_i_* and *∑_j_* denote the covariance matrices of the two classes *i* and *j*. The JMD is a parametric criterion with the values ranging from 0 to 2: separability is excellent when the values exceed 1.9, favorable when the values are between 1.4 and 1.9, and poor when the values are lower than 1.4. For a specific study site, the JMD from all possible pairwise classes was calculated and averaged to represent the overall separability of salt marsh vegetation, namely, the overall JMD (OJMD, Equation (6)).

(6) $$OJMD=\frac{\sum_{i\neq j}^{n}JMD_{ij}}{n}$$

where *n* is the number of all possible pairwise classes for a specific study site.

In addition, we introduced the random forest algorithm to obtain classification maps of salt marsh vegetation, which were also used to further support the separability analysis. The random forest is an ensemble classifier that uses multiple decision trees to make a prediction and has been proven to provide high accuracy with low bias, making it widely applicable in the classification of vegetation in coastal areas [8,51,52]. The holdout cross-validation method was introduced to select 50% of all samples to build a random forest classifier, the accuracy of which was verified using the remaining 50%. The overall accuracy (OA) was primarily used as the most direct indicator. Additionally, the quantity disagreement (QD) and allocation disagreement (AD) were also introduced since these enable a more thorough evaluation of the classification results [53]. On the basis of a specific confusion matrix, each element *p_ij_* in *i* row and *j* column indicates the proportion classified as class *i* but belonging to class *j* in actuality; the QD and AD can be calculated using Equations (7) and (8).

(7) $$QD=\frac{\sum_{i=1}^{n}\left|\sum_{j=1}^{n}p_{ji}-\sum_{j=1}^{n}p_{ij}\right|}{2}$$

(8) $$AD=\sum_{i=1}^{n}\min\left(\sum_{j=1}^{n}p_{ji}-p_{ii},\sum_{j=1}^{n}p_{ij}-p_{ii}\right)$$

## 4. Results

### 4.1. Characteristics of the VI Time-Series

**Figure 5:**
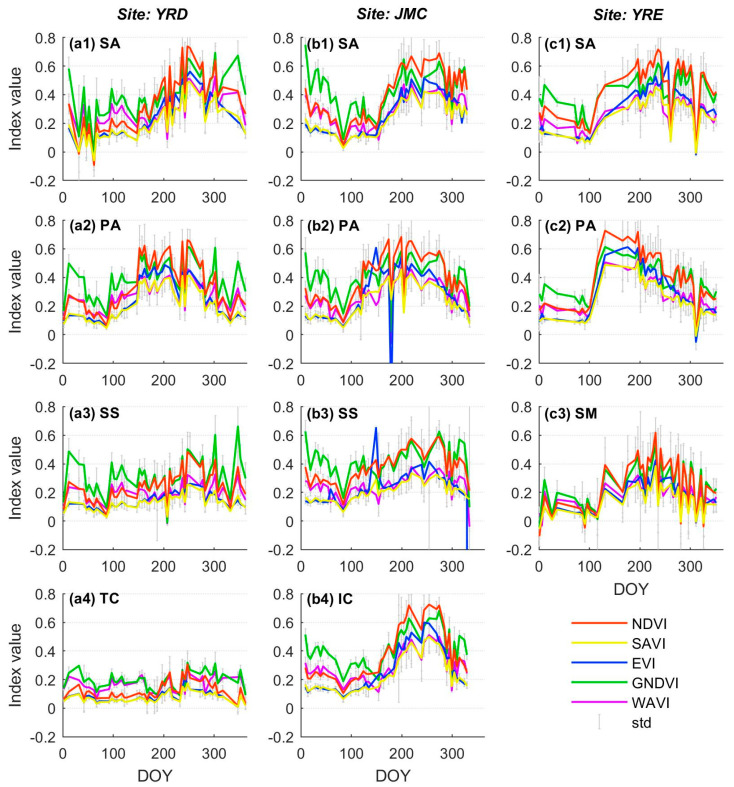
Trends and associated fluctuations for the different VI time series corresponding to different salt marsh vegetation types. Each sub-figure within the same column corresponds to different salt marsh vegetation types within the same site. Note: SA = *S. alterniflora*, PA = *P. australis*, SS = *S. salsa*, TC = *T. chinensis*, IC = *I. cylindrica*, and SM = *S. mariqueter*.

Figure 5 highlights the trends and fluctuations of the different VI time series as reflected by the change in the average value from each class of the salt marsh vegetation samples. In general, a certain degree of differentiation in the VI time series is observable for the different salt marsh vegetation types and represents a critical prerequisite for accurate vegetation classification. For example, a lagged phenological cycle particularly during the senescence season, is evidenced by *S. alterniflora* at all three study sites (Figure 5(a1–c1)), whilst an advanced phenological cycle, characterized by an early green-up and senescence season, is evidenced by *P. australis* (Figure 5(a2–c2)). Besides, the peak value for tall and dense plants (e.g., *S. alterniflora*, *P. australis*) is significantly higher than that for sparsely distributed vegetation such as *S. salsa* and *S. mariqueter* (Figure 5(a3–c3)).

Concerning the same class of salt marsh vegetation, the trend for various VI time series is similar, and the differences primarily manifest in terms of time series amplitude and stability. Compared with the NDVI time series, the characteristics of the other VI time series can be summarized as follows. The amplitude of the GNDVI time series is close to that of the NDVI time series during the vegetation growing season (March to November) but significantly higher than that of the other VI time series during the dormant season (December to February) during which most of the conspicuous fluctuations appear (e.g., Figure 5(a2,a3)). The amplitude of the EVI time series is lower than that of the NDVI time series throughout the whole phenological cycle. Crucially, this index is especially sensitive to noise, as evidenced by either local maximum (e.g., *S. salsa* in the JMC, DOY: 149, Figure 5(b3)) or minimum outliers (e.g., *P. australis* in the JMC, DOY: 179, Figure 5(b2)). In contrast, the SAVI time series is more stable, as the deviations affected by noise are relatively small, especially during the dormant season (e.g., Figure 5(b4,c2)). In terms of amplitude, the SAVI is generally consistent with the EVI time series (also lower than the NDVI time series), but is slightly lower during the growing season. The amplitude of the WAVI time series is similar to that of the NDVI time series during the dormant season, yet closer to the SAVI time series during the growing season (e.g., Figure 5(b1,c2)). Similar to SAVI, the stability of WAVI is also stable since the deviations in the time series are relatively small.

### 4.2. Fitting Accuracy for the VI Time-Series-Based Curve Fitting Models

**Figure 6:**
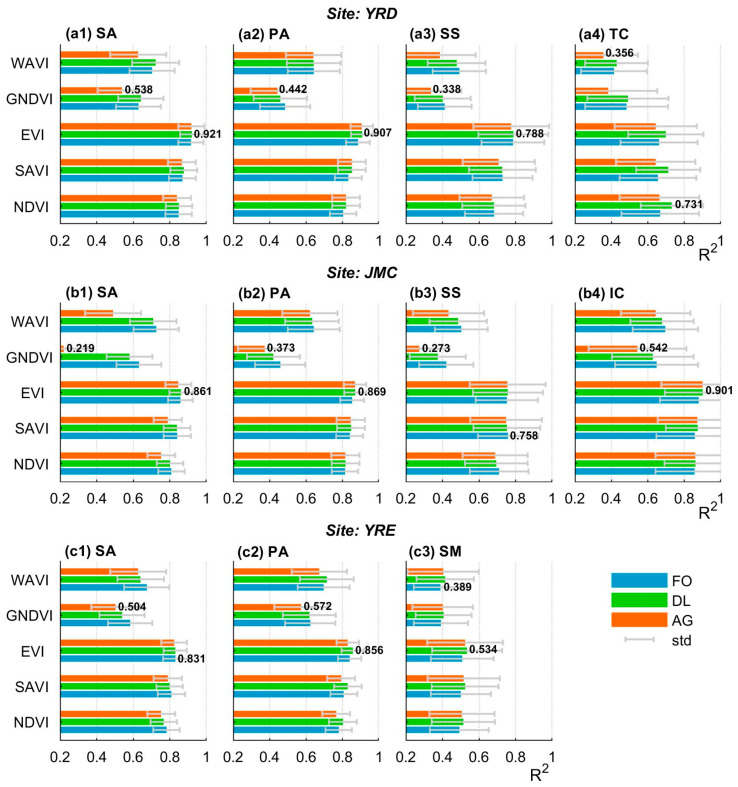
Fitting accuracy of the VI time series-based curve fitting model. Each sub-figure within the same row corresponds to different salt marsh vegetation types within the same site, whilst the two numerical labels for each sub-figure denote the maximum and minimum fitting accuracies. Note: SA = *S. alterniflora*, PA = *P. australis*, SS = *S. salsa*, TC = *T. chinensis*, IC = *I. cylindrica*, and SM = *S. mariqueter*; for the curve fitting model, AG = Asymmetric Gaussian, DL = Double Logistic, and TF = Two-term Fourier.

Generally, the differences in fitting accuracy among the VI time series are much larger than those generated by the curve fitting methods (Figure 6). For any given curve fitting method, the fitting accuracy of the EVI, SAVI, and NDVI time series is uniformly higher than that for the WAVI and GNDVI time series with a confidence level higher than 95% (i.e., *p* < 0.05, Table 4). This indicates that the former may better describe the VI time series, which performs particularly well during the vegetation growing season, rather than the dormant season.

Notably, the EVI time series achieves the highest fitting accuracy (average: 0.802) in 9/11 cases emerging from within each class of salt marsh vegetation at the three study sites. This accuracy is 0.028 higher than that obtained using the followed SAVI time series, and their difference is significant (*p* < 0.05) for *S. alterniflora* and *P. australis* at the three study sites (see Table 4). In contrast, the GNDVI time series has the lowest fitting accuracy (average: 0.482) in 9/11 cases. Such inferiority in fitting accuracy is very significant (*p* < 0.05) for almost all salt marsh vegetation types except *T. chinensis* in the YRD and *S. mariqueter* in the YRE. The remaining two cases belong to the WAVI time series, indicating that the latter two VI time series are unsuitable for curve fitting. Concerning the curve fitting method, the DL method exhibits the highest fitting accuracy in 9/11 cases, followed by the TF method. Nevertheless, except for *T. chinensis* in the YRD, the advantage of the DL method is not significant (*p* > 0.05) in terms of the average fitting accuracy compared to the TF method (see Table 5). In contrast, the AG method yielded a significantly (*p* < 0.05) low fitting accuracy of nearly all cases (10/11), especially for *S. alterniflora* in the JMC, where the fitting accuracy of the GNDVI time series reached only 0.219, which is significantly lower than that obtained using both the DL and TF methods (Figure 6(b1)). These data highlight the necessity of combining both the EVI time series and the DL method (i.e., EVI-based DL model) as this achieves the highest fitting accuracy (average: 0.830 for 8/11 cases), whilst in contrast, the GNDVI coupled with the AG model is deemed highly unsuitable (average fitting accuracy: 0.422 for 9/11 cases).

### 4.3. Saltmarsh Vegetation Separability Analysis

**Figure 7:**
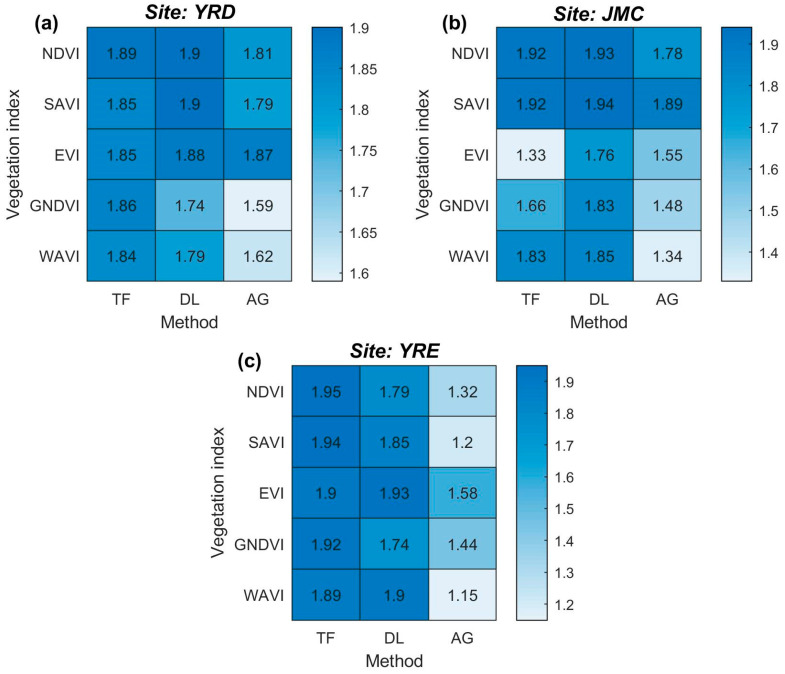
Salt marsh vegetation separability for the different VI time series-based curve fitting model measured by the overall Jeffries–Matusita distance (OJMD). (**a**–**c**) The OJMD for different VI time-series-based curve fitting models in the YRD, JMC, and YRE, respectively. Bluer cells equate to a higher degree of separability achieved by the model. Note: (i) the labeled value denotes the OJMD; and (ii) the curve fitting model, AG = Asymmetric Gaussian, DL = Double Logistic, and TF = Two-term Fourier.

The six phenological metrics (i.e., BV, MV, SOS, EOS, ROI, and ROD) were extracted from each VI time series-based curve fitting models and used in order to calculate salt marsh vegetation separability (Figure 7). The performance of salt marsh vegetation separability is shown to be highly related to both the time series stability and the accuracy of the fitting curve. The SAVI and NDVI time series are the most effective VI time series for separating the different salt marsh vegetation types, with consistently high OJMD scores in all three study sites. The SAVI and NDVI time series are highly stable, which is reflected in the OJMD scores >1.80 (except when combined with the AG method in the YRE) and thereby rank in the top three VI time series. In contrast, the performance of the EVI time series fluctuates significantly. In the YRD and YRE, the average OJMD scores reach 1.87 and 1.80, respectively, ranking in the top two for the same site (Figure 7a,c). However, this score declines rapidly in the JMC, especially for the EVI time series when coupled with the TF model which exhibits the lowest separability out of all the models (i.e., OJMD = 1.33, Figure 7b). For the GNDVI and WAVI time series, the OJMD scores usually rank in the last three of the VI time series, indicating that they are relatively unsuitable for salt marsh vegetation discrimination.

In terms of the curve fitting method, the DL and TF methods are suitable for discriminating between salt marsh vegetation types in the northern and southern study sites, respectively. Specifically, four out of the top five OJMD scores obtained from each study site were generated using either the DL or the TF method. However, the proportions of the DL and the TF methods change from 3:1 to 2:2 for the YRD and JMC study sites, respectively (Figure 7a,b), but finally reach 1:4 in the YRE (Figure 7c). The performance of the AG method is shown to deteriorate from north to south (e.g., the average OJMD score decreases from 1.74 to 1.34). Generally, the combinations of the SAVI and NDVI time series and the DL and TF curve fitting methods are superior in relation to distinguishing salt marsh vegetation. However, for northern areas (e.g., the YRD and JMC), the SAVI/NDVI-based DL model is highly recommended, whilst for southern areas (e.g., the YRE), the SAVI/NDVI-based TF model potentially yields better results.

**Figure 8:**
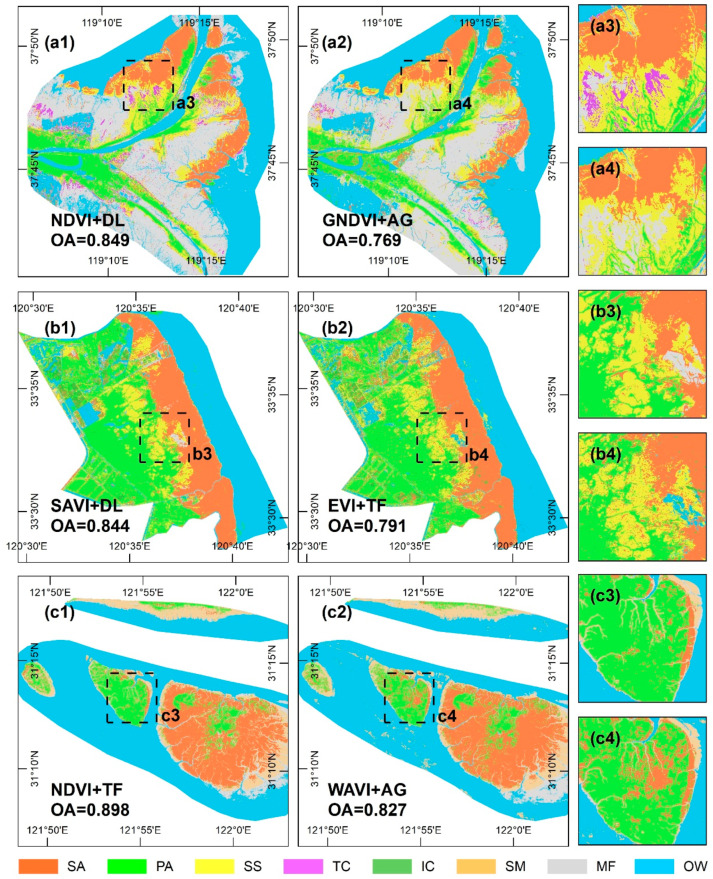
Classification maps for salt marsh vegetation corresponding to different VI time series-based curve fitting models. (**a1**–**c1**) Classification maps corresponding to the models with high separability, (**a2**–**c2**) classification maps corresponding to the models with low separability; (**a3**–**c3**) and (**a4**–**c4**) enlarged classification maps concentrating on the main differences from high separability and low separability models. Note: For salt marsh vegetation, SA = *S. alterniflora*, PA = *P. australis*, SS = *S. salsa*, TC = *T. chinensis*, IC = *I. cylindrica*, SM = *S. mariqueter*, MF = mudflat, and OW = open water. Note that for integrity of classification mapping, additional MF and OW samples were introduced, but OA calculation only involved salt marsh vegetation.

**Figure 9:**
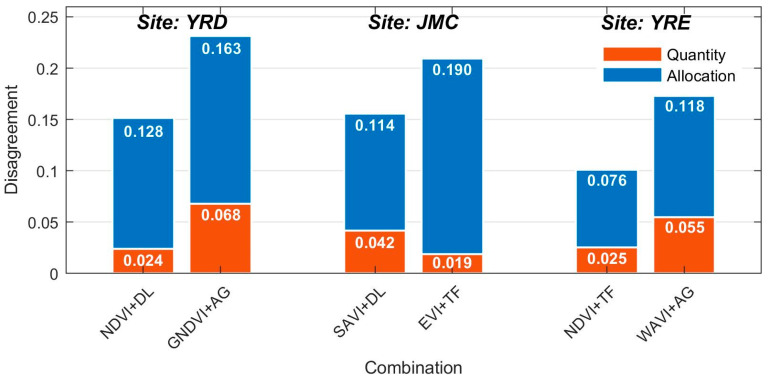
Quantity disagreement and allocation disagreement corresponding to the models with high and low separability for each study site.

Figure 8 presents the salt marsh vegetation classification map corresponding to the VI time series-based curve fitting model with the maximum and minimum separability for each study site. The classification maps are shown to be highly consistent with the separability results and are therefore accurate. The classification maps corresponding to the models with high separability reliably depict the actual distribution of salt marsh vegetation species, as evidenced by the strong agreement with data from the field surveys (Figure 8(a1–c1,a3–c3)). Conversely, the classification maps corresponding to the models with low separability yield a large number of omission errors (e.g., *T. chinensis*, see Figure 8(a2,a4)), commission errors (e.g., *P. australis* is misclassified as *S. alterniflora* in Figure 8(c2,c4)), and fragmentation (e.g., salt-and-pepper noise related to *S. alterniflora* is widespread in Figure 8(b2,b4)). More specifically, the allocation disagreement was shown to be the main source of error, having accounted for more than two thirds of the total agreement in each of the models (Figure 9). The most conspicuous difference between the models lies in the allocation disagreement in the JMC, which decreased by 0.076 when the high separability model was used. However, compared with the low separability model, the high separability model has a higher disagreement at this site. In terms of the YRD and YRE, the quantity and allocation disagreements for the model with high separability were uniformly lower than those for the model with low separability. In general, there is a 0.068 difference in the overall accuracy of the classification mapping between high- and low- separability models, representing a decrease in both the allocation disagreement (0.051) and quantity disagreement (0.017). As such, the choice of the VI time series-based curve fitting model has significant implications for the accuracy of salt marsh vegetation classification, highlighting the need for careful model selection.

## 5. Discussion

### 5.1. Impact of Complex Coastal Environments on VI Time-Series Analyses

**Figure 10:**
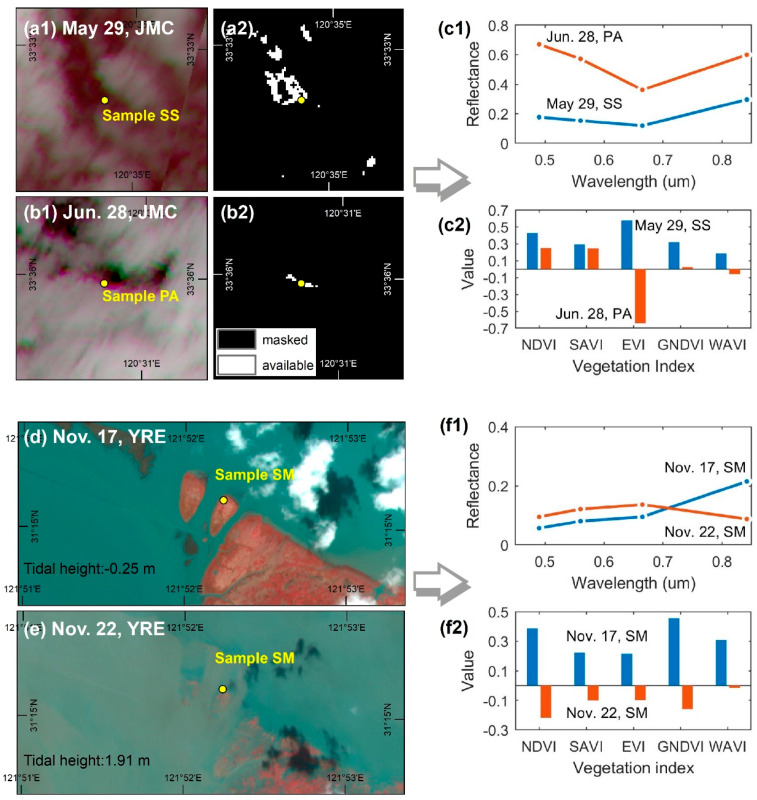
Impacts of coastal environments on the VI time series. Shown are two examples illustrating the errors arising from omitted clouds and shadows: (**a1**,**a2**) two Sentinel-2 MSI images (standard false color) with different acquisition dates on the left, and two corresponding SC maps on the right (**b1**,**b2**); (**c1**) spectral curves ranging from visible to near-infrared based for the two samples located in pixels containing omitted clouds/shadows; (**c2**) values for various VIs based on the two samples; (**d**,**e**) two Sentinel-2 MSI images (standard false color) acquired under different tidal flooding conditions; (**f1**) spectral curves based on the two samples under different tidal regimes; and (**f2**) values for various VIs based on the two samples.

Cloud cover in coastal areas is one of most notable causes of missing data during time series construction. Using a cloud detection band (e.g., the SC map) as a countermeasure, we filtered out all pixels corresponding to clouds and shadows instead of only using cloud-free images. This approach has gradually been accepted in recent years for mid- to high-spatial-resolution time series construction [25,26,29]. However, some errors can still be observed in the current cloud detection band due to omitted clouds/shadows (Figure 10(a1,a2,b1,b2)). The omitted clouds/shadows substantially influence the blue and green bands of the vegetation spectrum (Figure 10(c1)), leading to instability in the VI time series. The EVI was found to be most sensitive to such errors, probably because its calculation involves the blue band and uses a multiplier (7) to amplify the effect. This sensitivity is expressed as alternating local maximum and minimum outliers, and the difference between them is quite large (Figure 5(b2,b3) and Figure 10(c2)). Predictably, these outliers significantly obscure the general EVI time series trend, decrease the precision of phenological metrics, and ultimately weaken the performance of the vegetation classification. Notably, the outliers of the EVI time series appear less conspicuous in the YRE and YRD, but with the caveat that more samples fall within pixels with omitted clouds and shadows in the JMC than for the other areas where outliers are more widespread. Due to the random appearance of clouds and shadows within each image, the impacts of outliers on the EVI time series are sporadically distributed, leading to classification errors in the form of salt-and-pepper noises (Figure 8(b2,b4)). Similar patterns can also be observed in the GNDVI and WAVI time series, but the difference between the outliers is inferior to the EVI time series (Figure 10(c2)). In contrast, the SAVI and NDVI time series are relatively stable since their calculations involved neither blue nor green bands.

Periodic tidal flooding is another key factor affecting the efficacy of remote sensing applications in coastal areas. The extent of the impact of tidal flooding is comprehensively determined by factors such as tidal range, local topography, plant height, and density [28]. Generally, the larger the local tidal range, the lower the elevation of tidal flats, and the lower the height and density of the salt marsh vegetation, thereby rendering it more susceptible to tidal flooding. As a result, for the same class of salt marsh vegetation, the most obvious fluctuations in the time series usually occur in the YRE where the tidal range is highest (Figure 5(c1,c2)). At the same study site, the time series corresponding to pioneer salt marsh vegetation (*S. alterniflora* in the YRD and JMC; *S. mariqueter* in the YRE) fluctuates more frequently due to its lower elevation. *S. mariqueter* in particular appears to be the most vulnerable species to tidal flooding because of its lower plant height and density (Figure 5(c3) and Figure 10d,e). Previous studies have demonstrated that the main effect of tidal flooding and moist soil is embodied in a sharp decrease in the near-infrared (NIR) spectral reflectance for vegetation [54] (Figure 8(f1)), thus significantly attenuating a variety of VIs (Figure 10(f2)). This is also the main reason for occasional abrupt declines in the VI time series. However, different VIs demonstrate different degrees of attenuation to tidal flooding resulting from the introduction of additional spectral bands and adjustment factors. Consequently, the NDVI and GNDVI are found to be most sensitive to tidal flooding, while the SAVI and WAVI are relatively stable (Figure 10(f2)).

### 5.2. Impacts of Data Distribution on the Fitting Curve

**Figure 11:**
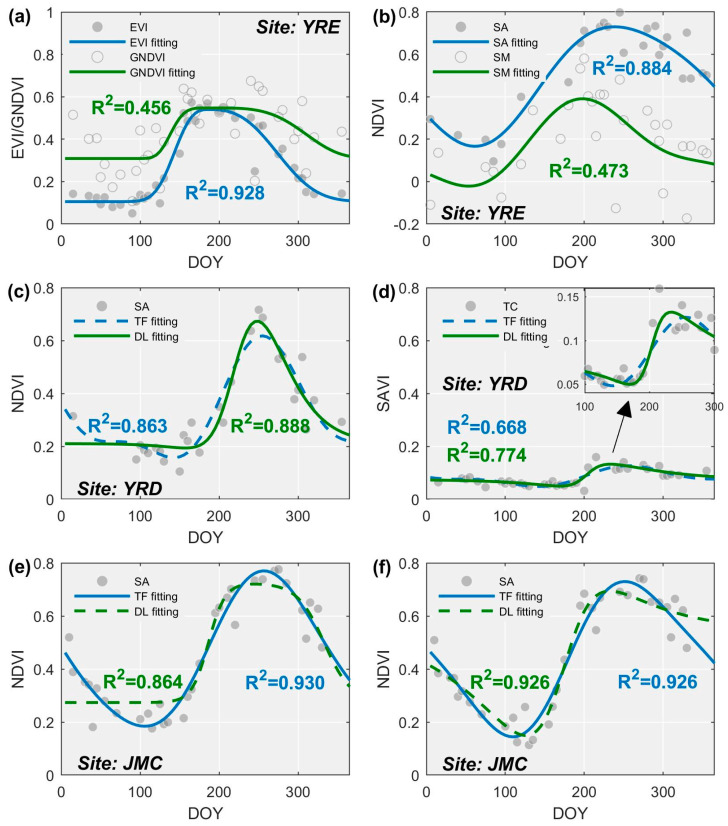
Impact of data distribution on curve fitting methods. (**a**) The impact of different VI time series (salt marsh vegetation: *P. australis*, fitting curve: AG); (**b**) the impact of different salt marsh vegetation (time series: NDVI, fitting curve: TF); (**c**) the advantage of the DL method for fitting time series in the YRD (salt marsh vegetation: *S. alterniflora*, time series: NDVI); (**d**) the advantage of the DL method for fitting time series of *T. chinensis* (time series: SAVI); (**e**,**f**) two examples illustrating the advance of the TF method for fitting time series in the JMC (salt marsh vegetation: *S. alterniflora*, time series: NDVI).

The suitability of the curve fitting method in relation to the available data distribution is also an important issue to consider as this varied markedly when different VI time series and fitting curve methods were combined for different classes of salt marsh vegetation (Figure 6). Our results demonstrate that the fitting accuracy from the EVI time series was commonly the highest, followed by the SAVI time series for the same class of salt marsh vegetation. This may be because the EVI and SAVI effectively eliminate NDVI saturation in high biomass regions. In contrast, the degree of fit between the GNDVI time series and the salt marsh vegetation is poor since it exhibits a “U-shaped” trend reflecting the transition from dormancy to the green-up season which cannot be modeled accurately by the presented curve fitting methods, especially the AG (Figure 11a). The VI time series for salt marsh vegetation less affected by tidal flooding (e.g., *P. australis*, *S. alterniflora*) tend to be better matched with the curve fitting methods. Salt marsh vegetation subjected to regular tidal inundation results in time series with major fluctuations, which often cannot be depicted by curve fitting methods (e.g., *S. mariqueter*, the fitting curve of which is underestimates actual abundance during the senescence season, Figure 11b).

Importantly, the most suitable curve fitting method is shown to switch from DL to TF from north to south (Figure 7), as determined by the distribution of available observations and the characteristics of the individual curve fitting method. For example, the YRE is located in the northern coastal region with a continental semi-arid climate, where salt marsh vegetation is usually covered by snow in winter but is less affected by cloudy weather in summer. As a result, there are few observations in the YRE from December to February, but many from June to August (Figure 2(a2) and Figure 11c). These characteristics of observation distribution match perfectly with the DL method because it needs enough data around the peak of the time series during the maturity season to accurately determine the range of each segment and amplitude of each function [42,43]. Although few observations render it impossible to reliably fit a trend to the time series data using the DL method even during the early green-up season, it can hardly represent the decrease of fitting accuracy since there were only a few observations available. Another notable advantage of the DL method lies in its ability to capture abrupt increases in the time series and accounts for why *T. chinensis* can be fitted accurately relative to other methods (Figure 6(a4) and Figure 11d). However, the coefficient of frequency (*w*) yielded by the TF method was often overestimated due to the lack of available observations during the dormant season (Figure 11c). Although the fitting accuracy of the TF method is slightly lower than that of the DL method, the time-related metrics (i.e., SOS and EOS) are likely to be heavily biased, thereby hindering subsequent discrimination of salt marsh vegetation.

In contrast, sufficient numbers of observations for the JMC and YRE can be obtained in winter but not in summer, owing to the heavy rainy season driven by a moist temperate/subtropical maritime monsoon climate. Besides, substantial fluctuations in VI time series are evident here during the summer due to the larger tidal range for these regions relative to the YRD, and which the DL method typically failed to capture. For example, for *S. alterniflora* exhibiting a lagged phenological cycle, the DL method presented either a prolonged flat peak or trough (Figure 11e), or a gradual decline during the senescence season as a trade-off for a relatively accurate description of the green-up season (Figure 11f). This is probably because the DL method is not suitable to describe minor fluctuations even though a natural system is being represented and enforces a single peak irrespective of the duration of the flatness at the peak or bottom [32]. In contrast, the TF method exhibits satisfactory performance with *S. alterniflora* because the Fourier function is flexible in that it alters the phase of function to adequately match the lags in phenology (Figure 11e,f).

Previous studies have typically evaluated the suitability of different curve fitting methods by using the fitting accuracy to then extract phenological metrics of vegetation [32,33]. However, fitting accuracy alone in relation to phenological metric-based vegetation classifications is insufficient, since this can describe different trends (Figure 11f). For example, in most cases, the fitting accuracy of the DL method is slightly higher than that of the FO method for the three study sites (Figure 6), but the separability of salt marsh vegetation in the JMC and YRE yielded by the FO method is better than that of the DL (Figure 6). Therefore, we suggest that the characteristics of the distribution within time series data need to be considered in future phenology-based applications.

## 6. Conclusions

The successful launch of the Sentinel-2 constellation satellite has enabled a range of remote sensing applications to be undertaken, including the remote sensing of vegetation in coastal regions. Utilizing satellite imagery from Sentinel-2, this study has presented a detailed time series analysis of salt marsh vegetation fluctuations recorded in three coastal sites in eastern China via a comparison of the vegetation index (VI) and several curve fitting methods—two key steps for time series construction. Five VIs (NDVI, SAVI, EVI, GNDVI, and WAVI) and three curve fitting methods (Asymmetric Gaussian-AG, Double Logistic-DL, and Two-term Fourier-TF) were applied to six dominant types of salt marsh vegetation. The main conclusions can be summarized as follows:

(1) The SAVI performed best for time series stability, as there was little noise introduced by tidal processes or cloud cover. In contrast, the EVI was found to be highly sensitive to the errors introduced by omitted clouds and shadows. (2) The EVI, SAVI, and NDVI time series were more suitable for the presented curve fitting methods than the WAVI and GNDVI. In particular, the DL curve fitting method commonly exhibited the highest fitting accuracy when applied in concert with the EVI time series to varied salt marsh vegetation. However, the TL method was also found to yield acceptable results. (3) The performance of salt marsh vegetation classification commonly depends on time series stability and fitting curve suitability. SAVI and NDVI time series were the most effective for discriminating salt marsh vegetation, especially combined with DL and TF methods (i.e., SAVI/NDVI-based DL/TF model). However, the EVI time series-based models usually demonstrated poor performance. The distribution of different climate-derived observations is another important factor for model selection, as the SAVI/NDVI-based DL model may be more favorable for high latitude regions with a continental climate. In contrast, the SAVI/NDVI based on the TF model is recommended for mid- to low latitude regions characterized by a monsoon climate.

## Figures and Tables

**Table 1:** Sample collection methods in relation to the classification of salt marsh vegetation.

Sample Collection Method	Description	Number of Samples
Field survey	Plots where the dominant salt marsh plant species occupied more than 80% of an area larger than 60 × 60 m were collected to ensure accurate vegetation classification. The geolocations of the plots were measured using a Real-Time Kinematic (RTK) GPS instrument to provide the positional accuracy suitable for the smallest sentinel-2 MSI pixel size of 10 m. Additional information such as plant species, growth stage, height and density was also recorded.	YRD: 65 JMC: 78 YRE: 43
UAV investigation	The UAV (Model: DJI M600, Shenzhen, China) was deployed at a height of ~50 m to save battery power and to obtain maximum ground coverage (i.e., >60 × 60 m). An RGB photo was taken where the dominant marsh plant species covered more than 80% of the field view. Ground control points were simultaneously measured using the RTK GPS to ensure positioning errors of the photos were less than 1 m.	YRD: 166 JMC: 117 YRE: 123
Google Earth interpretation	The textual and spectral characteristics of marsh plant species were obtained after projecting the field survey, and the UAV investigated plots in Google Earth snapshots. These characteristics were used to guide the interpretation of the marsh plant species in the random samples as prior knowledge.	YRD: 271 JMC: 331 YRE: 308

**Table 2:** Vegetation indices used in our study.

Name	Abbreviation	Formula	Reference
Normalized Difference Vegetation Index	NDVI	$\frac{\rho_{NIR}-\rho_{Red}}{\rho_{NIR}+\rho_{Red}}$	Rousel*,* et al. [37]
Soil-Adjusted Vegetation Index	SAVI	$\left(1+L\right)\frac{\rho_{NIR}-\rho_{Red}}{\rho_{NIR}+\rho_{Red}+L},\;L=0.5$	Huete [38]
Green Normalized Difference Vegetation Index	GNDVI	$\frac{\rho_{NIR}-\rho_{Green}}{\rho_{NIR}+\rho_{Green}}$	Gitelson and Merzlyak [39]
Enhanced Vegetation Index	EVI	$2.5\frac{\rho_{NIR}-\rho_{Red}}{\rho_{NIR}+6\rho_{Red}-7\rho_{Blue}+1}$	Huete*,* et al. [40]
Water-Adjusted Vegetation Index	WAVI	$\left(1+L\right)\frac{\rho_{NIR}-\rho_{Blue}}{\rho_{NIR}+\rho_{Blue}+L},\;L=0.5$	Villa*,* et al. [41]

**Table 3:** Phenological metrics used in our study and their calculation methods.

Phenological Metric	Calculation Method
Base Value (BV)	The minimum value of the fitting curve, which typically occurs in winter (e.g., December to February).
Maximum Value (MV)	The maximum value of the fitting curve, which typically occurs between late summer and early autumn (e.g., August to September).
Start Of Season (SOS)	The time when the fitting curve reaches 50% of the difference between BV and MV prior to the MV.
End Of Season (EOS)	The time when the fitting curve reaches 50% of the difference between the BV and MV subsequent to the MV.
Rate Of Increase at the beginning of the season (ROI)	The ratio of the difference in values ranging from 20% and 80% during the green-up season and the corresponding time difference.
Rate Of Decrease at the end of the season (ROD)	The ratio of the difference in values ranging from 20% and 80% during the senescence season and the corresponding time difference.

**Table 4:** Results of pairwise comparison among VI time series for *S. alterniflora* in the YRD based on Turkey HSD analysis.

Class 1	Class 2	Lower Confidence Interval	Estimate	Upper Confidence Interval	*p*-Value
NDVI	SAVI	−0.043	−0.025	−0.007	1.38 × 10^−3^
NDVI	EVI	−0.091	−0.073	−0.055	9.92 × 10^−9^
NDVI	GNDVI	0.225	0.243	0.261	9.92 × 10^−9^
NDVI	WAVI	0.143	0.161	0.180	9.92 × 10^−9^
SAVI	EVI	−0.066	−0.048	−0.029	9.93 × 10^−9^
SAVI	GNDVI	0.250	0.268	0.286	9.92 × 10^−9^
SAVI	WAVI	0.169	0.187	0.205	9.92 × 10^−9^
EVI	GNDVI	0.297	0.315	0.333	9.92 × 10^−9^
EVI	WAVI	0.216	0.234	0.252	9.92 × 10^−9^
GNDVI	WAVI	−0.099	−0.081	−0.063	9.92 × 10^−9^

**Table 5:** Results of pairwise comparisons among EVI-based curve fitting methods for *S. alterniflora* in the YRD based on Turkey HSD analysis.

Class 1	Class 2	Lower Confidence Interval	Estimate	Upper Confidence Interval	*p*-Value
TF	DL	−0.023	−0.006	0.012	7.27 × 10^−1^
TF	AG	−0.020	−0.002	0.015	9.42 × 10^−1^
DL	AG	−0.014	0.003	0.021	9.03 × 10^−1^

## References

[1] Barbier E.B., Hacker S.D., Kennedy C., Koch E.W., Stier A.C., Silliman B.R. (2011). The value of estuarine and coastal ecosystem services. Ecol. Monogr..

[2] Zedler J.B., Kercher S. (2005). Wetland resources: Status, trends, ecosystem services, and restorability. Annu. Rev. Environ. Resour..

[3] Isacch J.P., Costa C.S.B., Rodríguez-Gallego L., Conde D., Escapa M., Gagliardini D.A., Iribarne O.O. (2006). Distribution of saltmarsh plant communities associated with environmental factors along a latitudinal gradient on the south-west Atlantic coast. J. Biogeogr..

[4] Silvestri S., Defina A., Marani M. (2005). Tidal regime, salinity and salt marsh plant zonation. Estuar. Coast. Shelf Sci..

[5] Kirwan M.L., Megonigal J.P. (2013). Tidal wetland stability in the face of human impacts and sea-level rise. Nature.

[6] Schuerch M., Spencer T., Temmerman S., Kirwan M.L., Wolff C., Lincke D., McOwen C.J., Pickering M.D., Reef R., Vafeidis A.T. (2018). Future response of global coastal wetlands to sea-level rise. Nature.

[7] Carle M.V., Wang L., Sasser C.E. (2014). Mapping freshwater marsh species distributions using WorldView-2 high-resolution multispectral satellite imagery. Int. J. Remote Sens..

[8] Mahdianpari M., Salehi B., Mohammadimanesh F., Motagh M. (2017). Random forest wetland classification using ALOS-2 L-band, RADARSAT-2 C-band, and TerraSAR-X imagery. ISPRS J. Photogramm. Remote Sens..

[9] McCarthy M.J., Merton E.J., Muller-Karger F.E. (2015). Improved coastal wetland mapping using very-high 2-meter spatial resolution imagery. Int. J. Appl. Earth Obs. Geoinf..

[10] Beland M., Roberts D.A., Peterson S.H., Biggs T.W., Kokaly R.F., Piazza S., Roth K.L., Khanna S., Ustin S.L. (2016). Mapping changing distributions of dominant species in oil-contaminated salt marshes of Louisiana using imaging spectroscopy. Remote Sens. Environ..

[11] Hladik C., Schalles J., Alber M. (2013). Salt marsh elevation and habitat mapping using hyperspectral and LIDAR data. Remote Sens. Environ..

[12] Jia M.M., Zhang Y.Z., Wang Z.M., Song K.S., Ren C.Y. (2014). Mapping the distribution of mangrove species in the Core Zone of Mai Po Marshes Nature Reserve, Hong Kong, using hyperspectral data and high-resolution data. Int. J. Appl. Earth Obs. Geoinf..

[13] Fernandes M.R., Aguiar F.C., Silva J.M., Ferreira M.T., Pereira J.M. (2013). Spectral discrimination of giant reed (*Arundo donax* L.): A seasonal study in riparian areas. ISPRS J. Photogramm. Remote Sens..

[14] Ouyang Z.T., Gao Y., Xie X., Guo H.Q., Zhang T.T., Zhao B. (2013). Spectral discrimination of the invasive plant Spartina alterniflora at multiple phenological stages in a saltmarsh wetland. PLoS ONE.

[15] Albarakat R., Lakshmi V. (2019). Comparison of Normalized Difference Vegetation Index Derived from Landsat, MODIS, and AVHRR for the Mesopotamian Marshes Between 2002 and 2018. Remote Sens..

[16] Geng L.Y., Ma M.G., Wang X.F., Yu W.P., Jia S.Z., Wang H.B. (2014). Comparison of eight techniques for reconstructing multi-satellite sensor time-series NDVI data sets in the Heihe river basin, China. Remote Sens..

[17] Han X.X., Feng L., Hu C.M., Chen X.L. (2018). Wetland changes of China’s largest freshwater lake and their linkage with the Three Gorges Dam. Remote Sens. Environ..

[18] Sun C., Fagherazzi S., Liu Y.X. (2018). Classification mapping of salt marsh vegetation by flexible monthly NDVI time-series using Landsat imagery. Estuar. Coast. Shelf Sci..

[19] Sun C., Liu Y.X., Zhao S.S., Zhou M.X., Yang Y.H., Li F.X. (2016). Classification mapping and species identification of salt marshes based on a short-time interval NDVI time-series from HJ-1 optical imagery. Int. J. Appl. Earth Obs. Geoinf..

[20] Villa P., Bresciani M., Bolpagni R., Pinardi M., Giardino C. (2015). A rule-based approach for mapping macrophyte communities using multi-temporal aquatic vegetation indices. Remote Sens. Environ..

[21] Drusch M., Del Bello U., Carlier S., Colin O., Fernandez V., Gascon F., Hoersch B., Isola C., Laberinti P., Martimort P. (2012). Sentinel-2: ESA’s optical high-resolution mission for GMES operational services. Remote Sens. Environ..

[22] Filipponi F. (2019). Exploitation of sentinel-2 time series to map burned areas at the national level: A case study on the 2017 italy wildfires. Remote Sens..

[23] Falanga Bolognesi S., Pasolli E., Belfiore O.R., De Michele C., D’Urso G. (2020). Harmonized Landsat 8 and Sentinel-2 Time Series Data to Detect Irrigated Areas: An Application in Southern Italy. Remote Sens..

[24] Zhu Z., Wang S.X., Woodcock C.E. (2015). Improvement and expansion of the Fmask algorithm: Cloud, cloud shadow, and snow detection for Landsats 4–7, 8, and Sentinel 2 images. Remote Sens. Environ..

[25] Vrieling A., Skidmore A.K., Wang T., Meroni M., Ens B.J., Oosterbeek K., O’Connor B., Darvishzadeh R., Heurich M., Shepherd A. (2017). Spatially detailed retrievals of spring phenology from single-season high-resolution image time series. Int. J. Appl. Earth Obs. Geoinf..

[26] Villa P., Pinardi M., Bolpagni R., Gillier J.-M., Zinke P., Nedelcuţ F., Bresciani M. (2018). Assessing macrophyte seasonal dynamics using dense time series of medium resolution satellite data. Remote Sens. Environ..

[27] Vrieling A., Meroni M., Darvishzadeh R., Skidmore A.K., Wang T., Zurita-Milla R., Oosterbeek K., O’Connor B., Paganini M. (2018). Vegetation phenology from Sentinel-2 and field cameras for a Dutch barrier island. Remote Sens. Environ..

[28] Kearney M.S., Stutzer D., Turpie K., Stevenson J.C. (2009). The effects of tidal inundation on the reflectance characteristics of coastal marsh vegetation. J. Coast. Res..

[29] Zhu Z., Zhang J., Yang Z., Aljaddani A.H., Cohen W.B., Qiu S., Zhou C. (2020). Continuous monitoring of land disturbance based on Landsat time series. Remote Sens. Environ..

[30] Awty-Carroll K., Bunting P., Hardy A., Bell G. (2019). Using Continuous Change Detection and Classification of Landsat Data to Investigate Long-Term Mangrove Dynamics in the Sundarbans Region. Remote Sens..

[31] Guha S., Govil H. (2020). An assessment on the relationship between land surface temperature and normalized difference vegetation index. Environ. Dev. Sustain..

[32] Atkinson P.M., Jeganathan C., Dash J., Atzberger C. (2012). Inter-comparison of four models for smoothing satellite sensor time-series data to estimate vegetation phenology. Remote Sens. Environ..

[33] Hird J.N., McDermid G.J. (2009). Noise reduction of NDVI time series: An empirical comparison of selected techniques. Remote Sens. Environ..

[34] Zeng L., Wardlow B.D., Xiang D., Hu S., Li D. (2020). A review of vegetation phenological metrics extraction using time-series, multispectral satellite data. Remote Sens. Environ..

[35] Chung C.-H. (2006). Forty years of ecological engineering with Spartina plantations in China. Ecol. Eng..

[36] Gomez C., Dharumarajan S., Féret J.-B., Lagacherie P., Ruiz L., Sekhar M. (2019). Use of sentinel-2 time-series images for classification and uncertainty analysis of inherent biophysical property: Case of soil texture mapping. Remote Sens..

[37] Rouse J.W., Haas R.H., Schell J.A., Deering D.W. Monitoring Vegetation Systems in the Great Plains withERTS. Proceedings of the Third Earth Resources Technology Satellite—1 Symposium.

[38] Huete A.R. (1988). A soil-adjusted vegetation index (SAVI). Remote Sens. Environ..

[39] Gitelson A., Merzlyak M.N. (1994). Quantitative estimation of chlorophyll-ausing reflectance spectra: Experiments with autumn chestnut and maple leaves. J. Photochem. Photobiol. B: Biol..

[40] Huete A., Justice C., Van Leeuwen W. (1999). MODIS vegetation index (MOD13). Algorithm Theor. Basis Doc..

[41] Villa P., Bresciani M., Braga F., Bolpagni R. (2014). Comparative assessment of broadband vegetation indices over aquatic vegetation. IEEE J. Sel. Top. Appl. Earth Obs. Remote Sens..

[42] Jonsson P., Eklundh L. (2002). Seasonality extraction by function fitting to time-series of satellite sensor data. IEEE Trans. Geosci. Remote Sens..

[43] Beck P.S., Atzberger C., Høgda K.A., Johansen B., Skidmore A.K. (2006). Improved monitoring of vegetation dynamics at very high latitudes: A new method using MODIS NDVI. Remote Sens. Environ..

[44] Zhu Z., Woodcock C.E., Olofsson P. (2012). Continuous monitoring of forest disturbance using all available Landsat imagery. Remote Sens. Environ..

[45] White K., Pontius J., Schaberg P. (2014). Remote sensing of spring phenology in northeastern forests: A comparison of methods, field metrics and sources of uncertainty. Remote Sens. Environ..

[46] Zhang X., Friedl M.A., Schaaf C.B., Strahler A.H., Hodges J.C., Gao F., Reed B.C., Huete A. (2003). Monitoring vegetation phenology using MODIS. Remote Sens. Environ..

[47] Tamhane A.C. (1977). Multiple comparisons in model I one-way ANOVA with unequal variances. Commun. Stat. Theory Methods.

[48] Tukey J.W. (1949). Comparing individual means in the analysis of variance. Biometrics.

[49] Eklundh L., Jönsson P. (2017). Timesat 3.3 with Seasonal Trend Decomposition and Parallel Processing—Software Manual.

[50] Padma S., Sanjeevi S. (2014). Jeffries Matusita based mixed-measure for improved spectral matching in hyperspectral image analysis. Int. J. Appl. Earth Obs. Geoinf..

[51] Amani M., Salehi B., Mahdavi S., Brisco B. (2018). Spectral analysis of wetlands using multi-source optical satellite imagery. Isprs J. Photogramm. Remote Sens..

[52] van Beijma S., Comber A., Lamb A. (2014). Random forest classification of salt marsh vegetation habitats using quad-polarimetric airborne SAR, elevation and optical RS data. Remote Sens. Environ..

[53] Pontius R.G., Millones M. (2011). Death to Kappa: Birth of quantity disagreement and allocation disagreement for accuracy assessment. Int. J. Remote Sens..

[54] O’Connell J.L., Mishra D.R., Cotten D.L., Wang L., Alber M. (2017). The Tidal Marsh Inundation Index (TMII): An inundation filter to flag flooded pixels and improve MODIS tidal marsh vegetation time-series analysis. Remote Sens. Environ..

